# Prevalence and genotype identification of *Toxoplasma gondii* in suburban rodents collected at waste disposal sites

**DOI:** 10.1051/parasite/2019027

**Published:** 2019-05-01

**Authors:** Vladimir Ivovic, Sandra Potusek, Elena Buzan

**Affiliations:** 1 Department for Biodiversity, Faculty of Mathematics, Natural Sciences and Information Technologies, University of Primorska 6000 Koper Slovenia

**Keywords:** *Toxoplasma gondii*, Rodents, Waste sites

## Abstract

To assess the prevalence of *Toxoplasma gondii* infection in native and commensal rodents as indicators of environmental pollution, we analyzed brain tissue from small mammals collected on legal and illegal waste sites in the Slovenian and Croatian parts of Istria. A total of 136 animals and five species of the family Muridae were analyzed: black rat (*Rattus rattus*), domestic mouse (*Mus musculus*), wood mouse (*Apodemus sylvaticus*), striped field mouse (*Apodemus agrarius*), and yellow-necked mouse (*Apodemus flavicollis*). Using quantitative Polymerase Chain Reaction (qPCR), *T. gondii* DNA was detected in four homogenized brain tissue samples (2.94%), from all of the analyzed species, except black rat. Out of these, two samples, domestic mouse (*Mus musculus*) and wood mouse (*Apodemus sylvaticus*) had sufficient DNA for genotyping of *T. gondii* isolates in which we demonstrated the presence of clonal type II using RFLP PCR with four markers (SAG1, SAG2, GRA6 and GRA7). Three of four infected animals (75%) were collected on dumpsites.

## Abstract (fr)

Pour évaluer la prévalence de l’infection à *Toxoplasma gondii* chez les rongeurs indigènes et commensaux en tant qu’indicateurs de pollution environnementale, nous avons analysé le tissu cérébral de petits mammifères collectés sur des décharges légales et illégales des parties slovène et croate de l’Istrie. Au total, 136 animaux et cinq espèces de la famille des muridés ont été analysés : rat noir (*Rattus rattus*), souris domestique (*Mus musculus*), mulot sylvestre (*Apodemus sylvaticus*), souris des champs à rayures (*Apodemus agrarius*) et souris à collier (*Apodemus flavicollis*). À l’aide de la PCR quantitative, l’ADN de *T. gondii* a été détecté dans 4 échantillons de tissu cérébral homogénéisés (2,94 %) appartenant à toutes les espèces analysées, à l’exception du rat noir. Deux de ces échantillons, de souris grise (*Mus musculus*) et mulot sylvestre (*Apodemus sylvaticus*), contenaient suffisamment d’ADN pour le génotypage des isolats de *T. gondii*, dans lesquels nous avons démontré la présence de clones de type II en utilisant la PCR RFLP avec quatre marqueurs (SAG1, SAG2, GRA6 et GRA7). Trois des 4 animaux infectés (75 %) ont été collectés sur des décharges.

## Introduction

*Toxoplasma gondii* (Apicomplexa: Coccidia) is an intracellular apicomplexan protozoan parasite with an extremely broad host range, infecting a wide array of warm-blooded animals, including humans, which makes it one of the most successful protozoan parasites globally [1, 2, 28, 29, 30, 31]. *Toxoplasma gondii* is the causative agent of toxoplasmosis, its associated disease, which has significant economic, veterinary and medical importance [2, 16, 28]. Toxoplasmosis is amongst the most prevalent parasitic zoonoses, as about 30% of the human population has been estimated to be infected [1, 17]. Although the infection causes only mild clinical symptoms in most of the affected, certain groups of people, such as pregnant women, immunocompromized patients and, rarely, immunocompetent patients, can be in greater risk for developing more serious clinical disease, which can lead to a variety of other medical complications [1, 17]. A newly acquired infection in pregnant women can be transferred through the placenta and could cause birth defects such as intellectual disability, blindness and epilepsy, or in some cases even death of the fetus [5, 16, 28].

Humans can become infected in different ways, most commonly by eating undercooked pork [16, 21]. Pigs are omnivorous animals and can thus catch and ingest wild rodents, which serve as important intermediate hosts, consequently consuming tissue cysts. Pigs can also become infected by drinking contaminated water or consuming food containing rodent or cat feces. This is an additional route of infection for humans [5, 6, 17, 20, 21].

Small mammals and rodents in particular are reservoirs or intermediate hosts of various human pathogens. It has been proven that different species of rodents play a significant role in the transmission of certain contagious diseases to other animals and humans [15]. Previous field and laboratory studies have shown that wild rats are one of the most important intermediate hosts for *T. gondii* [15]. Hence, rodent monitoring as well as control are key ways to reduce its prevalence in the wild [15].

The Mediterranean coast in Slovenia and Croatia is a densely populated region. As the coastal population grows and urbanises, it also generates waste. An extensive network of urban landscape and natural habitat covers the Istrian peninsula shared between Slovenia and Croatia [18]. Despite the existing infrastructure for dealing with waste, studies have revealed an extensive network of illegal waste sites [19] across Europe. These sites provide a ready source of nutrition and shelter for human-introduced rodents [4] that support the spread of pathogen vectors and non-native/invasive species [25] along the Mediterranean coast [7, 14]. The higher parasitism and infection rates of animals in illegal waste sites could be significantly associated with the change in the rodent communities due to potential increase of commensal rodents brought there by human waste [4]. Rodents (commensal and wild) at these sites are either more exposed or more susceptible to parasites or infections due to higher population density, which is a consequence of additional sources of nutrition and shelter at waste sites. Since waste sites could present optimal conditions for rodents to breed with abundant supplies of food, we were interested in whether such sites with higher commensal rodent population densities also affect the occurrence of *T. gondii,* by analyzing brain tissues of rodents living in legal and illegal waste disposal sites.

## Materials and methods

Permits to work with animals and animal tissues were issued by the Ministry of Culture of the Republic of Croatia (No. 532-08-01-01/1-11-03) and the Veterinary Administration of the Republic of Slovenia (No. 34401-36/2012/9).

**Figure 1:**
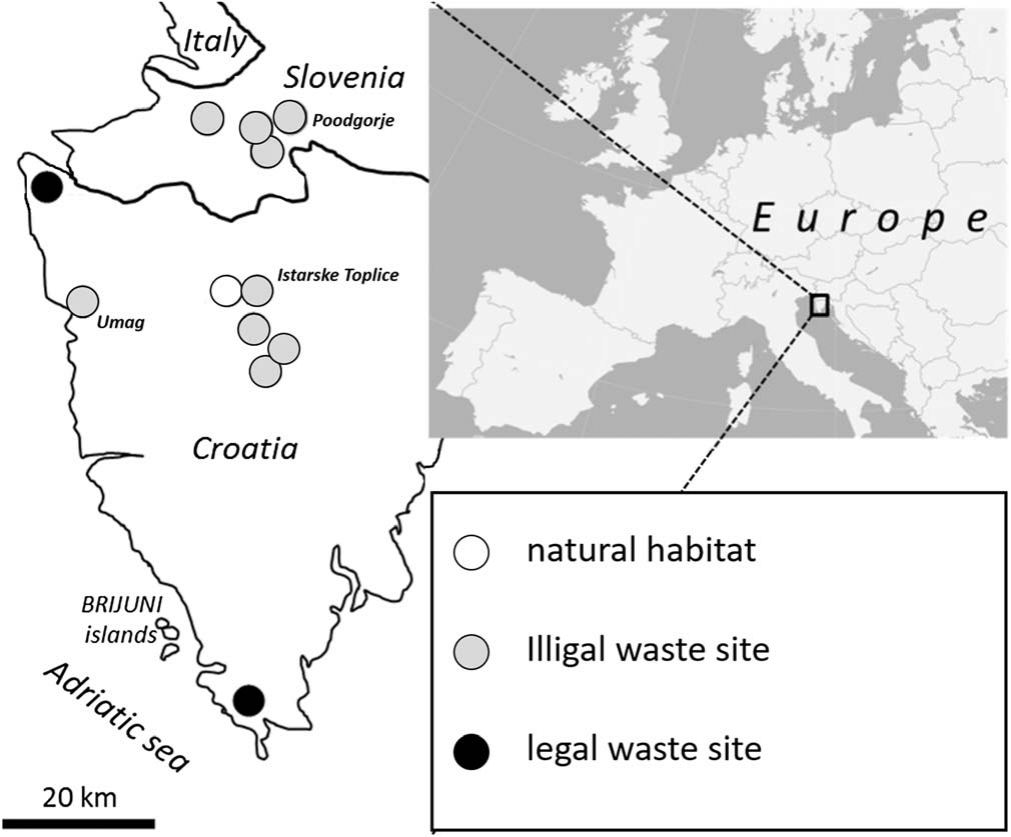
Waste and collection sites on the Istrian peninsula in Mediterranean Slovenia and Croatia.

We conducted our study in the coastal areas of Slovenia and Croatia, as they represent areas where faster urbanization caused by tourism has increased pressure on natural resources and wild animals. For this study, we selected 11 waste sites on the Istrian peninsula in Mediterranean Slovenia and Croatia according to availability and ownership issues, since some of the illegal waste sites were on private land (Fig. 1). All 136 animals were captured using live animal traps (LFATDG trap, 7.62 × 8.89 × 22.86 cm) during 2013 and kept frozen. For the purpose of this study, brains were obtained from all animals.

We used direct microscopic examination to check for *Toxoplasma* cysts in aliquots of homogenized brain tissue (Nikon Eclipse 55i, Nikon Corporation). The frontal part of the rodent brain including the olfactory bulb and orbital region were removed from the skull, homogenized by disposable micro tissue homogenizers, and 10 drops from each sample were microscopically examined.

To detect *T. gondii* DNA, aliquots of homogenized brain tissue from all rodents were checked for by quantitative Polymerase Chain Reaction (qPCR). All rodents were kept at −20 °C but, unfortunately we did not have any blood samples. Complete DNA from 100 μL of rodent brains was extracted with a QIAmp DNA mini kit (Qiagen GmbH), according to the manufacturer’s instructions. Extracted DNA was resuspended in 50 μL of nuclease-free water and stored at −20 °C. The *Toxoplasma* AF487550.1 gene (529-bp repetitive element), occurring up to 200–300 times in the *Toxoplasma* genome, was detected using HO1f 50-AGA GAC ACC GGA ATG CGA TCT-30 and HO2r 50-CCC TCT TCT CCA CTC TTC AAT TCT-30 primers with a Taqman probe (10 pmol/μL; 6FAM-ACG CTT TCC TCG TGG TGA TGG CG-TAMRA) [9, 22, 29]. Amplification was performed on a Roche LightCycler^®^ 96 System (F. Hoffmann-La Roche Ltd). To detect PCR inhibitors, DNA from a mimetic plasmid insert (1 pg/μL) was added in a second run to all samples [29]. Each amplification run included positive and negative controls. Samples were run in triplicate, and only animals that were positive in at least two assays were considered positive.

Genotyping of *T. gondii* DNA in the rodents’ brain tissue was performed by the PCR Restricted Fragment Length Polymorphism (RFLP) method based on four markers, including SAG1, SAG2, GRA6, and GRA7. For each marker, the PCR mixture consisted of 12.5 μL PCR Master Mix (Thermo Fisher Scientific, Inc.) (2×), 1 μL 10 μM (each) forward and reverse primer, 7.5 μL nuclease-free water, and 3 μL DNA extracted from the sample in a 25-μL reaction volume. Positive controls consisted of *T. gondii* type I, II and III reference strains, while nuclease-free water was used as a negative control. PCR products were digested with appropriate restriction enzymes for different markers. The PCR mixture for digestion consisted of 12 μL of nuclease-free water, 2.5 μL of buffer, and 0.5 μL of the appropriate restriction enzyme. Restriction products were visualized by electrophoresis using a 3% agarose gel stained with ethidium bromide. Estimation of fragment size was based on comparison to a 50-bp DNA ladder (Thermo Fisher Scientific, Inc.).

## Results

At total of 136 individuals of five species: *Rattus rattus*, *Mus musculus*, *Apodemus sylvaticus*, *A. flavicollis* and *A. agrarius*, from 11 different locations in the Istrian peninsula were tested for *T. gondii* DNA. Specific DNA was detected in four homogenized brain tissue samples (2.94%). Two of the four samples (*Mus musculus* and *Apodemus sylvaticus*) containing parasite DNA had enough DNA for genotyping of *T. gondii* isolates and demonstrated the presence of clonal type II using RFLP PCR with four markers, SAG1, SAG2, GRA6 and GRA7 (Table 1). Three of four infected rodents (75%) were collected on illegal waste sites, and one (*A. sylvaticus*) was collected at a near distance (10 m from the illegal waste site in the woods) in a natural habitat.

**Table 1:** qPCR and RFLP PCR results.

Species	Location	Tissue	PCR/DNA	SAG1	SAG2	GRA6	GRA7
*M. musculus*	Umag	Brain	+	II/III	II	II	II/III
*A. sylvaticus*	Podgorje	Brain	+	–	–	–	–
*A. flavicolis*	Podgorje	Brain	+	–	–	–	–
*A. agrarius*	Istr. toplice	Brain	+	II/III	II	II	II/III

By microscope examination, we found no cysts in any samples.

## Discussion

Seroprevalence is the best indicator of the presence of *T. gondii* in humans but in rodents this seems highly species-dependent [11]. Nonetheless, blood is not always available and we may use other methods to detect the presence of the pathogen in potential hosts. In rodents, cysts are distributed throughout the brain but more frequently are found in the olfactory bulb and orbital region [3]. For this reason, we focused our investigation on this very specific part of the brain in order to detect the presence of the pathogen DNA in the tissue using a qPCR technique.

The presence of the *T. gondii* DNA was not significantly prevalent amongst the five studied rodent species, *Rattus rattus*, *Mus musculus*, *Apodemus sylvaticus*, *A. flavicollis* and *A. agrarius* collected in waste disposal sites in Istria. Nevertheless, the diversity of infected hosts is intriguing, in four out of five recorded species, 2.94% in total were *Toxoplasma gondii* positive.

At waste sites, there are large quantities of organic waste that attract different vermin animals, particularly rodents. Results have shown that the transmission of other zoonotic pathogens between rodent species collected on waste sites, such as lymphocytic choriomeningitis virus, can be significantly increased by the presence of waste sites (53.33% in illegal waste sites *vs*. 17.39% in natural habitats) [7]. A study from Mexico showed that local rodent species have a relatively important role in the sylvatic cycle of *T. gondii*. [23]. Another study of urban rodents from Iran shown that 6% of all collected specimens were DNA positive in brain tissue detected by PCR [26].

Wild and commensal rodent species are generally common prey for both carnivores and omnivores, but there is a lack of information on how infected rodents may potentially contribute to the transmission of *T. gondii* in both domestic and sylvatic fauna. A study in the Piedmont Region (Northwestern Italy) has evaluated the prevalence of *T. gondii* infection in sympatric wild herbivores and carnivores. The results showed that the highest prevalence was recorded in red foxes (20.21%) followed by wild boars (16.19%), but we may assume that infected rodents can be a potential source of infection in these hosts. Nevertheless, results also showed that PCR detection of *T. gondii* DNA on skeletal muscle was significantly more sensitive than on the Central Nervous System (CNS) [8].

There are three main known clonal lineages of *T. gondii* (type I, II and III) found in Europe as shown by Howe and Sibley’s *T. gondii* population structure study [13]. One of the main goals of our study was also to obtain information regarding clonal lineages present in our pool of samples. We found that all of our infected samples were most likely clonal lineage II, which was not surprising, as this clonal type is predominant in Europe and North America [12, 15].

Although PCR-RFLP has a limited ability to distinguish between closely related isolates within a clonal line, performing analysis of four or five genetic markers is sufficient to discriminate types I, II and III, but also strains that have a genotype with two allele types at the same locus [15]. Clearly, if we analyze samples in which we can expect the presence of recombinant forms or atypical strains, it is better to include a larger number of markers: 9, 10 or even 12 [6, 27].

Previous results from the same area showed that improper waste management and dumping of organic waste attracts and brings to close encounter different wild and commensal rodent species [4]. Consequently, higher rodent population density may attract predators such as foxes, martens or domestic and wild cats. Arrival of commensal rodents to waste sites enables them to colonize the regions that are otherwise inhabited by native rodents, and gives them additional abilities for transmission of *T. gondii* from commensal rodents to native ones. We may hypothesize that humans indirectly affect the life cycle of pathogens in the natural environment through a remodeling prey-predator relationship and environmental contamination.

The classical life cycle of *T. gondii* relies on a prey-predator relationship and on environmental contamination. It is also dependent on populations of intermediate and definitive hosts, and on the level of predation between them [10, 24]. Garbage dumps occur mainly in rural areas where the population density of rodents and cats are already high or intermediate, including high predation rates. By introducing resources and changing the environment, humans may alter the structure of local rodent communities and make these areas particularly favorable for the transmission of *T. gondii*.

## Conclusion

The low prevalence of *T. gondii* at disposal sites in Istria suggests that rodents living in these areas do not represent a public health risk for people living close by. As this study was conducted only in one region of Mediterranean Slovenia and Croatia, it would be interesting to conduct similar studies in other parts of the country, and then compare the regions by the prevalence of *T. gondii.* This would also be beneficial in assessing the potential risks for humans and for finding out where to implement rodent control and/or where to take precaution measures in order to protect public health and wellbeing. Therefore, in future research we will pay more attention to the detection of pathogens also from the tissue samples.

For future studies, it may also be of interest to genotype more *T. gondii* DNA from rodent brain and tissue samples from different areas to assess which clonal type is the most prevalent in the countries, and to confirm or refute the hypothesis.
